# A Review of Potential Neuroimaging Biomarkers of Schizophrenia-Risk

**DOI:** 10.20900/jpbs.20230005

**Published:** 2023-05-26

**Authors:** Daniel Mamah

**Affiliations:** Department of Psychiatry, Washington University Medical School, St. Louis, MO, 63110, USA

**Keywords:** schizophrenia risk, psychosis risk, high risk, CHR, UHR, neuroimaging, structural, functional connectivity, diffusion imaging, PET, MR spectroscopy, ASL

## Abstract

The risk for developing schizophrenia is increased among first-degree relatives of those with psychotic disorders, but the risk is even higher in those meeting established criteria for clinical high risk (CHR), a clinical construct most often comprising of attenuated psychotic experiences. Conversion to psychosis among CHR youth has been reported to be about 15–35% over three years. Accurately identifying individuals whose psychotic symptoms will worsen would facilitate earlier intervention, but this has been difficult to do using behavior measures alone. Brain-based risk markers have the potential to improve the accuracy of predicting outcomes in CHR youth. This narrative review provides an overview of neuroimaging studies used to investigate psychosis risk, including studies involving structural, functional, and diffusion imaging, functional connectivity, positron emission tomography, arterial spin labeling, magnetic resonance spectroscopy, and multi-modality approaches. We present findings separately in those observed in the CHR state and those associated with psychosis progression or resilience. Finally, we discuss future research directions that could improve clinical care for those at high risk for developing psychotic disorders.

## INTRODUCTION

Schizophrenia and related psychoses are estimated to have a lifetime prevalence of about 3% and is associated with significant distress, functional disability, and decreased mortality [1]. Multiple studies have shown that earlier treatment of psychotic disorders results in improved long-term outcomes [2,3], and interventions during the prodrome can improve functional outcomes and potentially prevent illness onset [4–6]. Symptoms suggestive of the schizophrenia prodrome are sometimes seen in those at increased genetic risk, particularly first-degree relatives of schizophrenia probands. A recent meta-analysis found the risk of schizophrenia in those with one first-degree relative is about 7.7-fold higher, and over 11-fold higher with two first-degree relatives, compared to those without [7].

Over the last three decades, there have been efforts to characterize the psychosis prodrome prospectively. The clinical high-risk (CHR), otherwise referred to as ultra-high-risk, construct has been operationalized for research studies, and most commonly comprises of attenuated psychotic symptoms associated with functional decline and preserved insight [8–10]. Less prevalent CHR cases are based on brief intermittent psychotic symptoms or a syndrome of high genetic risk associated with functional decline. Among those meeting CHR criteria, an estimated 15–35% will develop a psychotic disorder within three years [11–16], which is greater than in first-degree relatives of probands.

Multiple neuroimaging studies of psychosis-risk populations have been conducted over the years; however, these have differed in the imaging modality used, design, and sample size, leading to inconsistent results. Brain findings in high-risk populations have often found to be intermediate between that of first-episode schizophrenia patients and the healthy controls [17]. Identifying reliable brain-based biomarkers—objectively measurable physical traits associated with a clinical condition that are replicable—could facilitate early intervention in CHR youth to improve outcomes.

This narrative review summarizes findings from neuroimaging studies of those at CHR, including single studies as well as meta-analyses, and systematic reviews across multiple modalities. Neuroimaging findings are separated into those observed at baseline and those associated with psychosis progression/conversion. The potential clinical utility of existing neuroimaging markers in those at risk for psychosis and potential limitations are discussed.

## METHODS

This paper is a narrative review of neuroimaging findings in individuals at clinical high risk (CHR) or psychosis, which is a comprehensive review of the available literature but is not exhaustive. The aim was to identify the largest and most representative neuroimaging studies in CHR populations, including most meta-analyses and systematic reviews. The search process to identify studies to include in the review involved:

1. Search through PubMed using the following relevant keywords and search criteria, with Boolean logic: “MRI”, “diffusion”, “structural”, “functional connectivity”, “positron emission tomography”, “magnetic resonance spectroscopy”, “arterial spin labeling”, “clinical high risk”, “ultra-high risk”, and “psychosis”.
2. Only neuroimaging studies involving CHR (or UHR) subjects at baseline or with psychosis conversion were considered for inclusion. Exclusions included studies focusing primarily on interventions, trait- or substance-related effects, cross-modality interactions, or highly novel methodologies.
3. All meta-analyses and systematic reviews associated with the above search terms were selected for inclusion. These studies were identified by specifying them under “article type” during the search process.
4. All studies involving diffusion imaging, functional connectivity, PET, and ASL were selected for inclusion. Additional studies were randomly selected to total at least thirty-five structural imaging, ten functional activation studies, and ten MRS studies.

## CLINICAL HIGH RISK FOR PSYCHOSIS

Neuroimaging findings presented here are those observed in CHR populations, without regard to conversion status. CHR studies have generally used one of two assessment instruments for case ascertainment, with similar diagnostic criteria: the Structured Interview of Psychosis-Risk Syndromes (SIPS) and the Comprehensive Assessment of At-Risk Mental States (CAARMS) [18].

### Gray Matter Structural Studies

The gray matter of the brain consists primarily of neuronal cell bodies, neuropil (dendrites and unmyelinated axons) and glial cells, and relatively few myelinated axons. Measures of gray matter structure in research studies are typically derived from T1-weighted anatomical MRI images [19]. Table 1 summarizes the multiple structural imaging studies involving cortical and subcortical gray matter in CHR youth [20–36]. While several published studies found no significant group differences between CHR and control youth [26,36–39], most studies have reported group effects, though the abnormal regions found in the CHR cases have been variable.

A voxel-based meta-analysis reported gray matter volume decreases in CHR subjects compared to controls in right STG and MFG; left precuneus and medFG; and bilateral parahippocampal/hippocampal regions and anterior cingulate [24]. Another voxel-wise meta-analysis found that antipsychotic naïve CHR subjects had gray matter reductions in regions spanning the right MTG and STG, in the right parahippocampus/hippocampus, in the left anterior cingulate and the right MFG [57]. A systematic review and meta-analysis of voxel-based morphometry studies found that genetic risk for psychosis was associated with gray matter volume reductions in the right cerebellum and left amygdala, while CHR was associated with decreased gray matter volumes in the SFG [58], generally small effect sized which did not survive statistical correction. A separate systematic review and meta-analysis reported larger pituitary volumes in CHR youth compared to the controls [45]. Additionally, a multi-center study found the CHR group had decreased the size of the frontal regions bilaterally [54].

To overcome the limitation of small sample sizes in many structural studies, the ENIGMA initiative pooled data from 31 international sites, including 3169 participants (1792 CHR and 1377 healthy controls), with MRI processed using harmonized protocols and analyzed within a mega-analysis and meta-analysis framework [41]. The study found widespread decreases in structural metrics in CHR individuals, with group effects of −0.13 (95% CI: −0.2–−0.06) for ICV, −0.18 (95% CI: −0.25–−0.11) for mean cortical thickness, and −0.18 (95% CI: −0.22–−0.08) for total surface area. Additionally, significant cortical thinning was found in 42 out of 68 regions, with effect sizes ranging from −0.09 to −0.18. The largest effect sizes (>−0.15) were found in the right ITG, MTG, STG, lateral occipital, and precuneus; left fusiform gyrus, IPL, and paracentral gyrus; and bilateral insula. Three of the 16 subcortical regions showed significant group effects (*d* = −0.1–−0.16) with CHR subjects showing the largest volumetric reduction in the right hippocampus. Eight of 68 surface areas showed significant group effects, with effect sizes generally lower (*d* < −0.12) than those found for cortical thickness.

One morphometric study investigating the shape of the hippocampus reported a relative inversion of the left ventral posterior hippocampus in CHR subjects compared to the controls [48].

**Figure 1.**
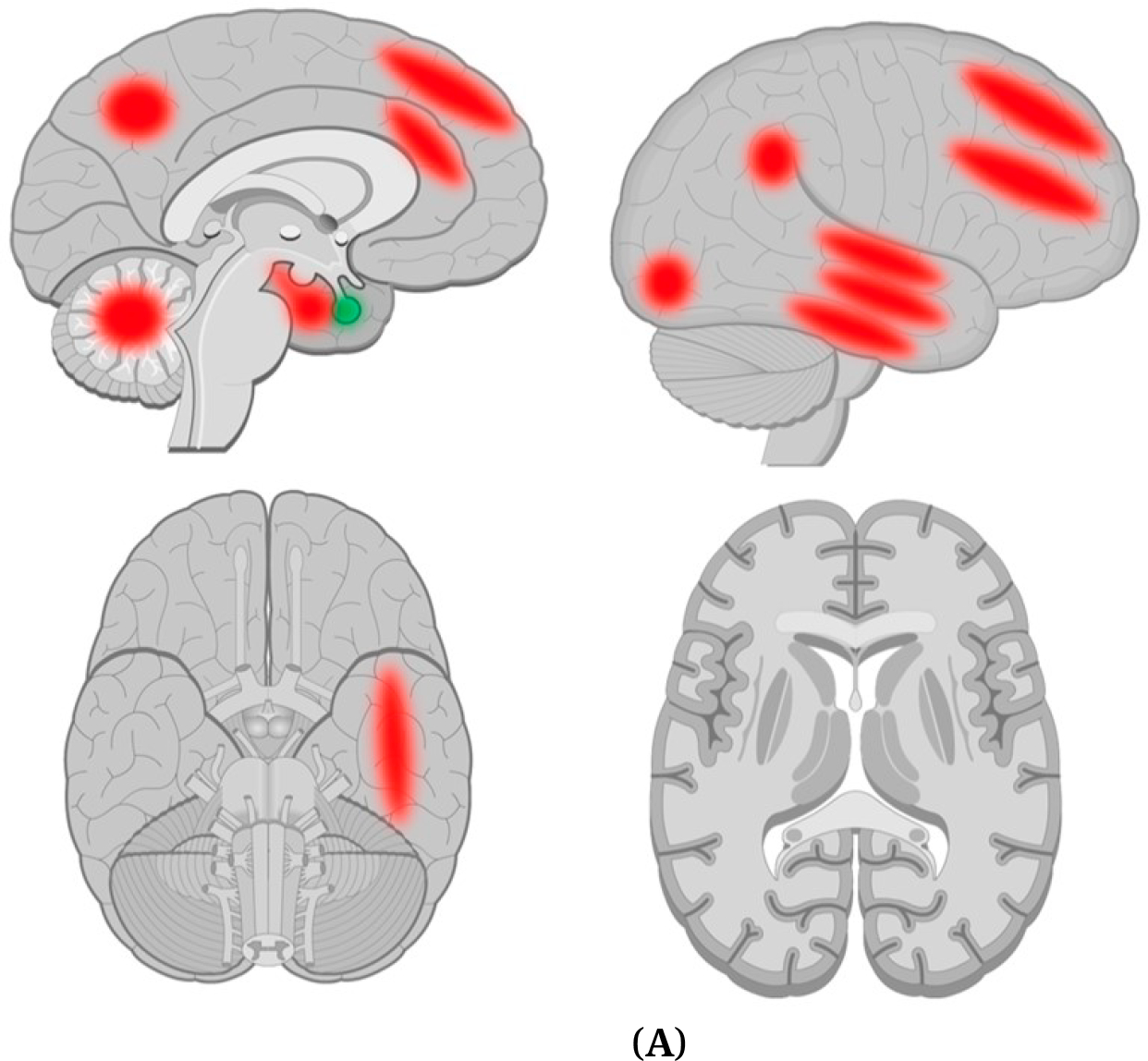

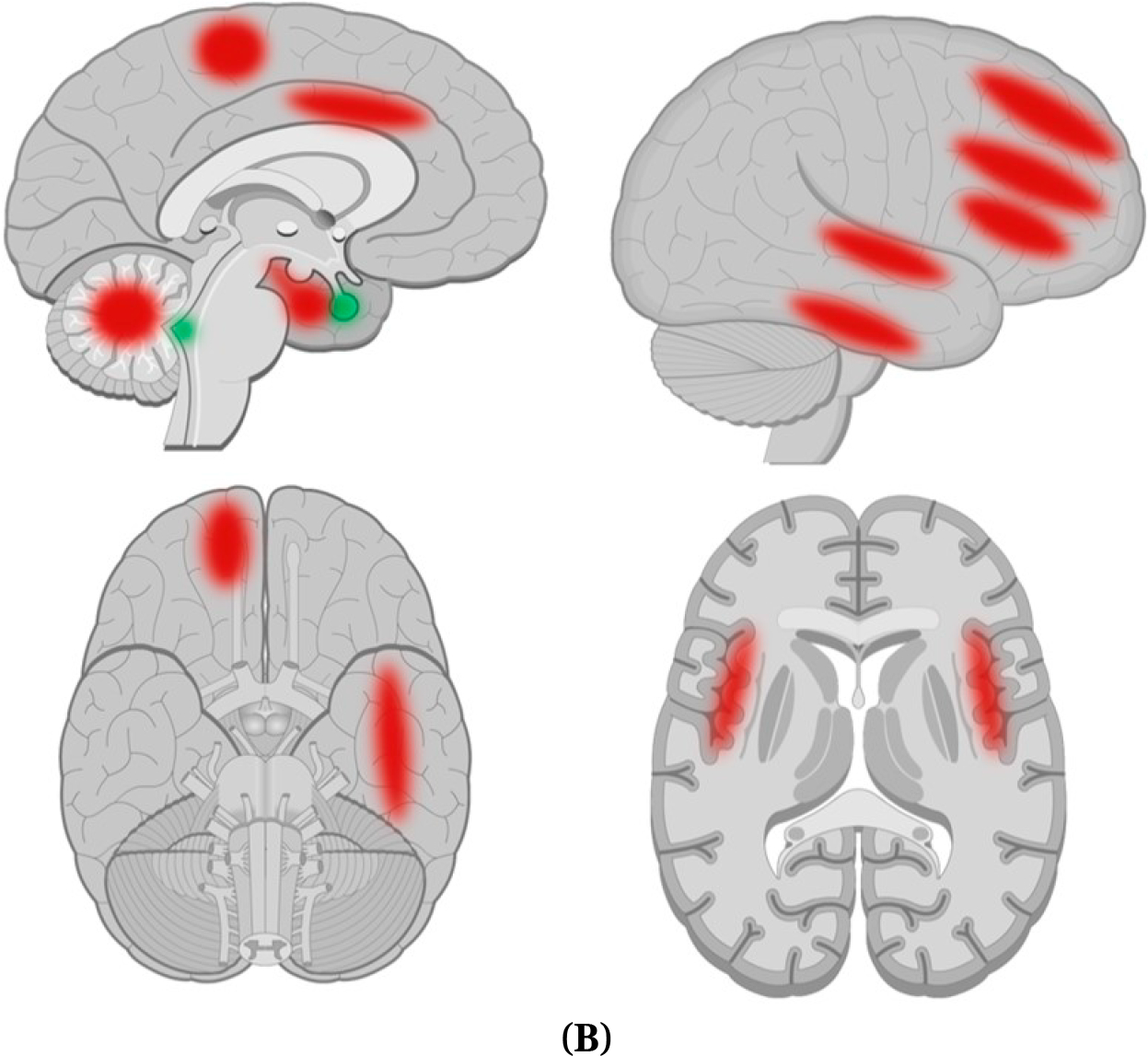
**(A) Brain regions found abnormal in structural gray matter studies of CHR compared to control subjects.** Red = Thinning or reduced volume. Green = Increased volume. **(B) Brain regions found abnormal in structural gray matter studies of CHR converters compared to CHR non-converters.** Dark red = Thinning or reduced volume. Green = Increased volume.*Brain images adapted from https://www.getbodysmart.com/.

Figure 1A shows pertinent brain regions found abnormal in the CHR structural studies reviewed.

### White Matter Structural Studies

T1-weighted anatomical MRI images are also used to investigate white matter structure. The white matter of the brain is comprised primarily of long-range myelinated neuronal axons, and relatively few cell bodies. There have been relatively few studies focusing on white matter structural abnormalities in the CHR state, as shown in Table 2. One study reported a volumetric decrease only in the right STG white matter in CHR subjects compared to a more widespread volumetric decrease in first-episode psychosis patients [59]. Findings from diffusion imaging studies, which identify white matter tract integrity, have been heterogeneous. A global reduction of fractional anisotropy (FA) has been reported in the CHR subjects [60], while others have found reduced FA in the cingulum bundle [61]. Reduced FA or increased mean diffusivity (MD) has also been reported in the SLF with CHR in two separate studies [62,63].

**Figure 2.**
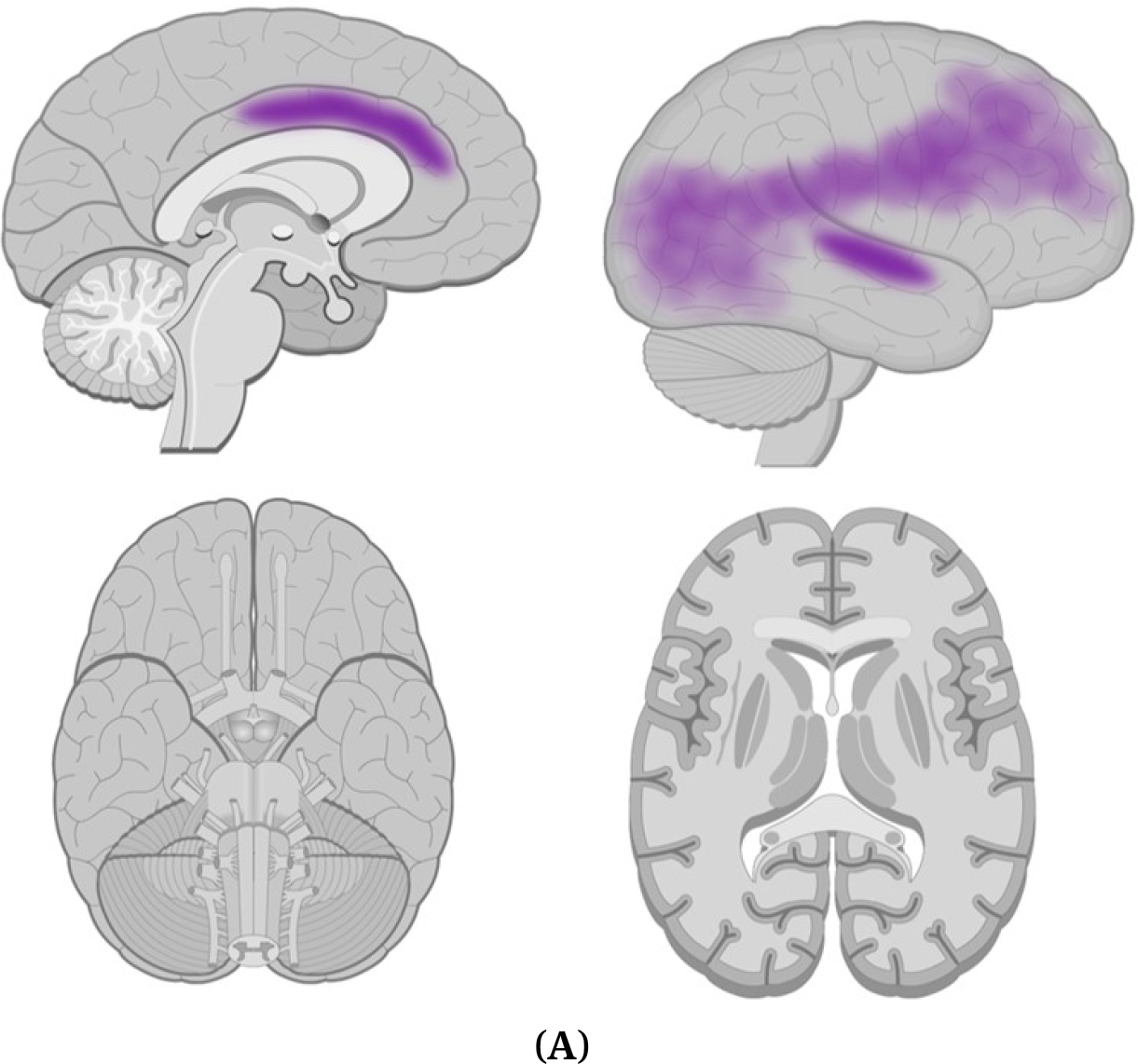

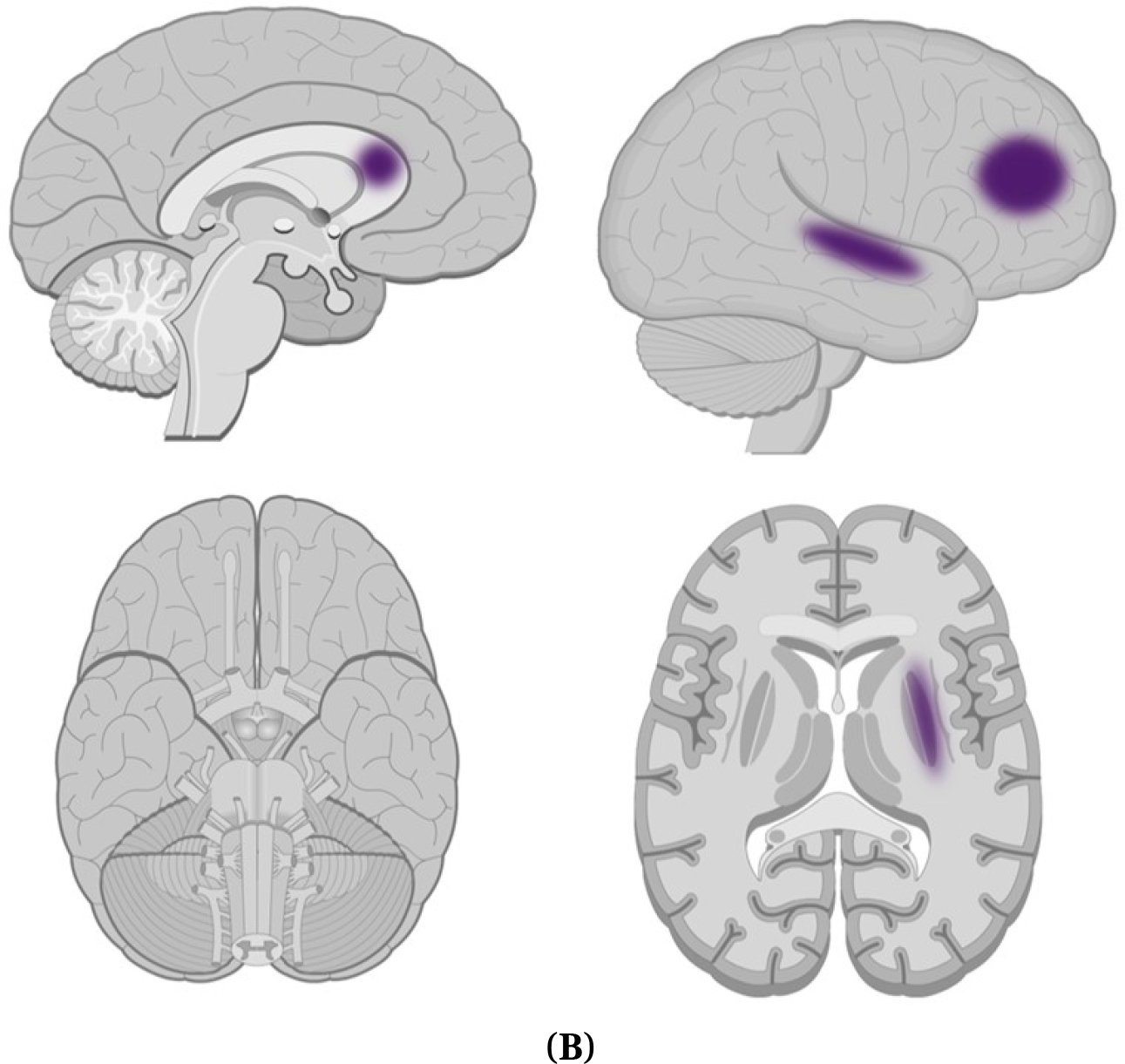
**(A)Brain regions found abnormal in white matter studies of CHR compared to control subjects.** Purple = Thinning or reduced volume. **(B)Brain regions were found abnormal in white matter studies of CHR converters compared to CHR non-converters.** Dark purple = Thinning or reduced volume. *Brain images adapted from www.getbodysmart.com.

Figure 2A shows brain regions found to be abnormal in the CHR structural studies reviewed.

### Functional Connectivity Studies

The temporal coincidence of spatially distant neurophysiological events can be measured using functioning magnetic resonance imaging, to determine regional interactions in the brain at a macro level. Studies using this method referred to as functional connectivity imaging, are most commonly done under resting state conditions [79].

Table 3 shows functional connectivity studies in CHR populations. CHR subjects have been reported to have widespread resting-state thalamocortical connectivity disruptions [80], involving hypoconnectivity of the thalamus with the PFC and the cerebellum, as well as hyperconnectivity between the thalamus and sensory-motor cortices. Others have reported baseline cerebellar dysconnectivity findings including increased cerebellar-DMN connectivity [81] and hyperconnectivity in the cerebello-thalamo-cortical circuitry in a large multicenter study [82]. CHR subjects have also been reported to maintain abnormally high DMN activity in a verbal working memory task [83] and during an emotion activation task to show increased activation in the amygdala and decreased activation in the vlPFC [84].

### Other Functional Magnetic Resonance Imaging Studies

Unlike functional connectivity imaging studies, which are based on finding a temporal correlation of spatial remote brain events, functional MRI can also be used to identify spatial activation in the brain, typically in association with a cognitive task [79]. A systematic review and meta-analysis found hypoactivation in the right precuneus, SFG, and right IFG in CHR [58]. A quantitative review reported that CHR subjects showed dysfunction in the right IPG and SFG; and in the left MFG and STG [107]. CHR subjects have also been reported to increase activation of the STG during a working memory task compared to controls in two studies [108,109], with decreased activation of frontoparietal regions in one study [109].

Arterial spin labeling (ASL) is a non-invasive MRI perfusion method based on changes in the net magnetization transfer of blood water used to measure the cerebral blood flow [110]. A study using ASL showed increased regional cerebral blood flow (rCBF) in the hippocampus, basal ganglia, and midbrain in CHR subjects compared to controls [111].

Functional imaging and ASL studies pertaining to the CHR state are shown in Table 4.

### Positron Emission Tomography Studies

Positron emission tomography (PET) is a minimally-invasive functional imaging method involving the use of intravenous radioactive substances (radiotracers) to visualize and measure specific metabolic or biochemical processes in the brain [120]. PET studies conducted in CHR cases are summarized in Table 5.

In CHR subjects, increased 18F-DOPA uptake has been found in the associative subdivision of the striatum [125,129] or the midbrain [128], suggesting that dopamine overactivity may predate the onset of schizophrenia.

Despite challenges with its interpretation, radiotracers for TSPO, an outer mitochondrial membrane protein associated with injury as well as microglial and astrocytic activation, have been considered markers of the neuroinflammation [133,134]. An elevated TSPO signal was found in the gray matter of CHR subjects when [^11^C]PBR28 was used as the ligand and was also correlated with the symptom severity [123], However, no increase in TSPO signal was found in CHR subjects when [^11^C]PK11195 radioligand was used [121], or in the DLPFC and the hippocampus when the [^18^F]FEPPA radioligand was used and controlled for rs6971 polymorphism [122].

### Magnetic Resonance Spectroscopy Studies

MRS is an analytical technique associated with magnetic resonance imaging used to determine the relative concentrations of a variety of biochemicals in the brain, and for monitoring brain metabolism in vivo [135]. Neuronal loss and active myelin breakdown have been estimated using the major peaks of the ^1^H-MRS spectrum, corresponding to N-acetyl aspartate (NAA), creatine (Cr), and choline (Cho) containing phospholipids. NAA is considered a non-invasive marker of neuronal health, while Cho is a marker of cell membrane turnover, generally elevated in demyelination, inflammation, and gliosis [136]. A decreased ratio of NAA to Cr (NAA/Cr) is considered a metabolic marker of neuronal or axonal loss or dysfunction.

Table 6 shows the few published MRS studies in CHR youth. One study reported a reduction in NAA/Cr and NAA/Cho ratios in the left frontal cortex and NAA/Cr in the anterior cingulate in CHR subjects compared to the controls [137], indicating a neuronal loss in these regions. Another study found increased NAA/Cr and Cho/Cr in the DLPFC in CHR subjects, which had been interpreted as indicative of the hypometabolism [138].

### Multimodal Neuroimaging Studies

Increased glutamate and cerebral blood volume (CBV) have been reported in the hippocampus of CHR subjects compared to the controls [147].

Combining MRS and PET [TSPO] in CHR subjects did not show a negative correlation between the anti-oxidant glutathione and TSPO in the mPFC which was seen in normal controls indicating an abnormal redox status in the high-risk group [148].

Another PET-MRS study found that in CHR subjects there was a negative association between GABA levels and the TSPO signal in the medial PFC [149].

### Machine Learning Applied to Neuroimaging Data

Machine learning has been used to increase the accuracy of individual-specific predictions and is based on multivariate analysis and pattern recognition. The support vector machine (SVM) is the most common type of machine learning method used in psychiatric neuroimaging studies. It learns first by training on a dataset of known outcomes and is later validated by applying it to an independent set.

SVM applied to structural MRI data has been reported to identify CHR with an accuracy of 72% (sensitivity of 68% and specificity of 76%) [150]. A multi-site study applying SVM to both gray and white matter structural MRI data as well as rs-fMRI data resulted in a classification accuracy of 90.8% for CHR subjects [151]. Another group trained a machine learning algorithm on healthy subjects’ gray matter volumes and estimated the ‘brain age’ [152]. They found an 0.64-year average brain age gap. Using cognitive data, other groups created an SVR-based age prediction model and found that CHR subjects had mean cognitive age gap estimates (CogAGE) of 4.3 years, which was associated with increased gray matter volume in temporal and frontal gray matter areas and diffuse patterns of white matter reductions [153].

fMRI data associated with working memory tasks separated CHR subjects from controls with a balanced accuracy of 76.2% (sensitivity 89.5% and specificity 63.2%) [154]. Another fMRI study found 88% sensitivity and 91% specificity focused on regional homogeneity summarizing functional connectivity between regions and their local neighbors [155].

## PSYCHOSIS PROGRESSION AND SYMPTOM CHANGE

Most outcome studies in CHR subjects report neuroimaging changes related to conversion to schizophrenia or psychosis, which constitutes the majority of what is summarized in the text following and also in Tables 1–6. However, clinical outcomes in those at CHR are variable, with symptom progression occurring in some people without meeting the criteria for a psychotic disorder. Other outcomes include remission, no change, and the development of a mood disorder.

### Gray Matter Structural Studies

A voxel-based meta-analytic study showed that CHR converters had smaller right ITG and STG compared to non-converters [24]. Another meta-analysis reported decreased gray matter volume in prefrontal, cingulate, insular, and cerebellar cortices in the CHR converters [55]. A large multisite study found greater loss in the right SFG, MFG, and medial orbitofrontal cortices in converters compared to non-converters [37]. Another multicenter study found less gray matter in the left parahippocampal cortex in CHR youth who converted to psychosis compared to those who did not [54]. Conversion in CHR has been also associated with hippocampal atrophy in the CA1 region [147]; reduced insular volume [56]; and decreased right medial and lateral temporal cortices and inferior frontal cortex, and decreased cingulate cortex bilaterally [29].

Lateral ventricular enlargement is one of the most notable structural findings in schizophrenia, however, the few studies investigating ventricular size in the CHR did not find a difference between converters and non-converters [51]. One study however reported that converters had larger third ventricles than non-converters [37]. Outside of the ventricles, the only notable structure reported enlarged in CHR converters is the pituitary, as reported in a meta-analysis [45].

In the ENIGMA study, conversion to psychosis was associated with the lower thickness of the left fusiform, right superior temporal, and bilateral paracentral cortices (mean Cohen *d* = −0.22; 95% CI, −0.35 to 0.10), which were similar to cortical thickness findings in ENIGMA studies of schizophrenia [41], as well as those with 22q1.2 deletion syndrome and a psychotic disorder. The small to modest effect size differences accounted for approximately 1% of the variance in CHR+/−comparisons. The study also found that the left paracentral gyrus showed a significant group by age interaction. Between ages 12–16, control participants showed a steeper decline in the thickness of the left fusiform cortex compared to the CHR group. Right-sided regions trended similarly, though did not meet statistical significance. This suggests that the normative pruning process during adolescence may be impeded in some regions in CHR populations.

Symptom progression in CHR subjects in CHR subjects have been associated with reduced cortical thickness in the right lateral and medial temporal cortex and left insular cortex [49], and inversion of the left ventral posterior hippocampus using shape analysis [48]. CHR youth who remitted also did not have a decline in the volume of their hippocampal CA1 region compared to those who did not [46]. Another study found that CHR youth who were resilient showed larger baseline cortical thickness of frontal, temporal, and parietal cortices and volumes of the nucleus accumbens and corpus callosum than those who were non-resilient [156].

Figure 1B shows brain regions found to be abnormal in CHR converters compared to non-converters in the structural studies reviewed.

### White Matter Structural Studies

Reduced thickness of the anterior genu of the corpus callosum has been reported in CHR converters compared to non-converters [78].

Conversion has also been associated with decreased FA in the left frontal lobe [75] and decreased FA in medial frontal lobes, left putamen, and left superior temporal lobe [76]. However, an absence of group differences between converters and non-converters has also been described [77]. These are depicted in Figure 2B.

Reported white matter structural predictors of psychosis progression have included increased FA in the thalamomotor tract [101] and increased FA in inferior frontal-occipital fasciculus, anterior thalamic radiation, SLF, and corticospinal tracts [69]. Regarding resilience markers, improvement in positive symptoms has been associated with increased integrity of the corpus callosum [73].

### Functional Connectivity Studies

Thalamocortical connectivity disruptions at rest have been found in those who convert to psychosis [80]. Reported impairments have included hypoconnectivity of the thalamus with the PFC and cerebellum, and hyperconnectivity between the thalamus and sensory-motor cortices [80].

Conversion has also been linked to a progressive efficiency decrease in the DMN and increased network diversity[91]; dissimilar functional network organization [93]; aberrant structural covariance in salience, executive control, auditory, and motor networks [157]; altered midbrain-prefrontal connectivity [104]; altered cingulate topological features [105]; altered connectivity in dorsal anterior cingulate cortex, mid-cingulate cortex, supplementary motor area, and mesial SFG [100]; and cerebello-thalamo-cortical hyperconnectivity [82]. Regarding resilience factors, improvement in clinical outcomes and symptoms have been associated with higher between-network connectivity (among language, dorsal attention, cerebellar, sensorimotor, and salience networks) and a more typical modular connectome organization [92].

Task-based functional connectivity markers predicting illness course in CHR have included increased activation in bilateral PFC, brainstem (midbrain/basilar pons), and left hippocampus and greater midbrain-prefrontal cortex connectivity during verbal fluency [104]; greater activation in STG, caudate and left IFG during a language processing task [115]; and less activation in PFC, precuneus and temporal lobes during a theory of mind task [158].

### Other Functional Magnetic Resonance Imaging Studies

CHR converters have also been found to have a positive association between age and activation in the DLPFC, IFG, frontal eye fields, and SFG during a verbal working memory task, findings which may reflect compensatory mechanisms [114].

Reported ASL-based markers predicting illness course in CHR subjects have included increased hippocampal [116], pallidum [117], and striatum rCBF [118].

### Positron Emission Tomography Studies

Conversion to psychosis has been associated with increased striatal dopamine synthesis [127], increased dopamine levels [126], as well as increased 18F-DOPA uptake in the midbrain [104].

### Magnetic Resonance Spectroscopy Studies

Baseline glutamate, myoinositol, and creatinine levels are higher in CHR subjects who converted to psychosis in the hippocampus [143]. Higher hippocampal glutamate levels were also associated with poor functional outcomes in CHR individuals [143]. Higher baseline glutamate levels have also been found in the associative striatum of converters compared to non-converters [145]. In addition, thalamic glutamate levels at baseline have been reported to be lower in a CHR non-remission group compared to those who remitted [146].

Proton MRS studies have reported a large effect size reduction of neuronal density (decreased NAA/Cho) and increased membrane turnover (increased Cho/Chr) in anterior cingulate in converters compared to non-converters [137].

### Multimodal Neuroimaging Studies

A correlation between GABA concentration in the medial PFC and hippocampal rCBF have been found in CHR converters compared to non-converters [116].

### Machine Learning Applied to Neuroimaging Data

Based on a machine learning algorithm from healthy subjects’ gray matter volumes, the brain gap was found to be increased to 1.59 years in younger (12–17 years old) CHR converters, from that seen in the baseline CHR group [152].

An SVM algorithm trained on structural MRI data from CHR and healthy control subjects found a balanced accuracy of 84.2% (sens: 81% and spec: 87.5%) in classifying converters vs. non-converters [159]. A similar but slightly lower accuracy was found in a follow-up study [160].

Using machine learning on structural imaging data was found to predict ‘good’ or ‘poor’ GAF outcomes with an accuracy of 82% [161]. Support vector regression analysis was used to predict along a continuous scale, reporting the highest correlation of 0.42 between long-term functioning and subcortical volumes [162]. One-year social and role functioning outcomes were predicted using structural MRI variables (accuracy 76.2%), clinical variables (accuracy 16.9%), and combined variables (accuracy 82.7%), suggesting that combining modalities can increase prediction accuracy [163]. In this study, medial prefrontal and temporal-parietal-occipital gray matter volume (GMV) reductions and cerebellar and dorsolateral prefrontal GMV increments were regions that had predictive value [163].

## DISCUSSION

We present a narrative review of neuroimaging findings in youth at clinically high-risk for psychosis, with an emphasis on major studies in the field. The review underscores the diversity of neuroimaging findings in the CHR population, as well as the heterogeneity of results across studies. Nevertheless, the pattern of brain abnormalities observed is generally less extensive or attenuated compared to that seen in schizophrenia. Structural imaging studies usually indicate shrinkage of gray matter, most notably the frontal and temporal cortices in those at high risk for psychosis. These abnormalities are thought to reflect the cumulative result of impaired maturational process, including proliferation, myelination and synaptic pruning [164–167]. It is also influenced by environmental factors, such as drug use and psychosocial stress [168,169], the latter often accentuated by a heightened hypothalamic-pituitary-adrenal stress response in this population [170]. Reduced white matter tract integrity and functional dysconnectivity have also been reported in CHR subjects, suggesting that like schizophrenia, cortical thinning is associated with impaired regional communication. Results from the limited positron emission studies conducted in CHR populations suggest that increased striatal dopamine uptake and cortical inflammation may underlie the psychosis-risk state.

Neuroimaging biomarkers have the potential to improve psychiatric care and have potential clinical utility in several areas. Firstly, they could help predict psychosis progression, which would guide clinical decision-making such as determining appropriate interventions. Antipsychotic medications, often held in CHR patients to prevent unnecessary side effects, may be required earlier in patients with brain profiles predictive of developing schizophrenia. Similarly, the presence of resilience markers may minimize the need for pharmacological interventions and suggest a greater role for psychotherapeutic approaches. Secondly, neuroimaging biomarkers may be useful in monitoring treatment effectiveness on brain structure and function, particularly when the accuracy of reported symptoms is uncertain. In this scenario, a clinician may decide on alternative interventions if brain abnormalities worsen, even with reported symptomatic improvement. Thirdly, neuroimaging tools may be useful in medication selection if the effects of specific medications on brain structure and function can be reliably determined. Finally, neuroimaging can identify brain-based biotypes across CHR populations which could improve the validity of diagnostic classifications. Such a reconceptualization of the clinical construct could generate relatively homogenous CHR populations which are more amenable to clinical trials using novel interventions.

Several challenges remain in establishing clinically useful neuroimaging biomarkers for high-risk patients. A general limitation to using neuroimaging markers in psychiatric populations is that of inadequate sensitivity [171]. Existing CHR neuroimaging studies aggregate data from groups of subjects for analysis, with a high degree of variability within groups and considerable overlap between the distributions of the two groups. This limits the generalizability of reported group findings to individuals. The specificity of neuroimaging findings is another limitation, as reported neuroimaging abnormalities in the CHR often share considerable similarities to those seen in other psychiatric disorders, including depression, anxiety disorders, or neurodevelopmental disorders. Multimodality imaging approaches may be more specific to psychotic disorders, though relatively few such studies have been conducted in psychosis-risk individuals. Results of neuroimaging studies can also differ based on the specific control populations included, since CHR syndromes usually present with other types of psychopathologies. Designs using healthy control populations recruited from the general community are inherently unable to disentangle group differences due to psychosis-specific processes or co-occurring psychopathology [172]. Help-seeking control designs, on the other hand, have the advantage of better accounting for co-morbidities among the CHR population, and group differences are more likely to indicate psychosis-specific processes [172]. The reported brain studies on psychosis progression are limited by high attrition and relatively low rates of conversion, usually within a 2- or 3-year period, limiting the accuracy of resulting predictive markers. Furthermore, cross-study differences in sample size, age, illness onset, help-seeking status, medication history, substance use and assessment tools used can also influence results. Therefore, large, multi-center studies are required to increase statistical power, however, fully harmonizing acquisition protocols across sites are not always attainable. In considering the utility of individual neuroimaging modalities, it is also important to consider their limitations. Newer neuroimaging methods continue to be developed, including those probing tract inflammation [173], individual-specific functional networks [174], T2-relaxation properties of brain tissue [175], and novel radiotracers [176,177], which may further improve clinical stratification of high-risk youth. Ultimately, integrating data from multiple modalities could prove the most useful.

The wealth of existing neuroimaging studies of CHR subjects has advanced our understanding of mechanisms involved in psychosis development. While the role of imaging findings in illness etiology is still not completely understood, it may be time to begin investigating the benefits of incorporating potential neuroimaging biomarkers of psychosis risk into clinical practice.

## Figures and Tables

**Table 1.** Structural gray matter neuroimaging studies in CHR and psychosis conversion.

Author/year	Modality	Study Type	Age range (mean)	Case *n*	Country Other	Abnormalities
Luna et al. 2022 [40]	Structural (gray)	SR/MA	26.3	2801	na	↓sup frontal (n.s.)
ENIGMA 2021 [41]	Structural (gray)	MA	21.1	1792	na ENIGMA	↓cortical thickness in CHR ↓thickness of fusiform, sup temp and paracentral in conv.
Merritt et al., 2021 [42]	Structural (gray)	SR	na	2473^a^	na	accelerated cortical decline
Del Re et al. 2021 [22]	Structural (gray)	SS	18.8	92	China	none in CHR ↓thickness in banks STS, Heschl’s, pars triang with conv.
Fortea et al., 2021 [43]	Structural (gray)	MA	22.3	1148	na	none in CHR ↓R temporal, ACC, paracingulate w conv.
Zikidi et al. 2020 [36]	Structural (gray)	SS	21.7	114	Scotland	none
Ding et al., 2019 [44]	Structural (gray)	SR/MA	na	743	na	↓ vol superior frontal, R rectus ↑vol median cingulate, R fusiform, L STG, R thalamus
Chung et al. 2019 [21]	Structural (gray)	SS	12–35	378	US/Canada NAPLS	↓cortical volume ↓area of ACC, prefrontal, parahipp in younger converters
Kwak et al. 2019 [28]	Structural (gray)	SS	20.6	74	South Korea	↓prefrontal, inf parietal
Tomyshev et al. 2019 [33]	Structural (gray)	SS	20.4	30	Russia	↓frontal, temp, parietal thickness
Saunders et al. 2019 [45].	Structural (gray)	SR/MA	13–29	432	na	↑pituitary ↑pituitary with conv.
Sakuma et al. 2018 [21]	Structural (gray)	SS	21.0	45	Japan	none
Takayanagi et al. 2017 [32]	Structural (gray)	SS	22.6	73	Japan	↑area of ACC ↓thickness of ACC with conv.
Ho et al. 2017 [46]	Structural (gray)	SS	21.2	93	Singapore	none in hippocampus in CHR Shrinkage in CA1 with symptom persistence and conv.
Walter et al. 2016 [47]	Structural (gray)	SR/MA	21.2	939	na	slight ↓R hippocampus in CHR
Dean et al. 2016 [48]	Structural (gray)	SS	18.9	38	US	↓hippocampus, inversion in L ventral hipp
Cannon et al. 2015 [37]	Structural (gray)	SS	12–35	274	US/Canada NAPLS	no baseline differences ↓rate of loss in R sup frontal, mid frontal, med orbitofr in converters
Klauser et al. 2015 [26]	Structural (gray)	SS	21.5	69	Singapore	none conv. ↑whole brain vol in conv.
Tognin et al. 2013 [49]	Structural (gray)	SS	23.9	40	England	thinning of L insula, R temporal with symptom progression
Nordholem et al. 2013 [50]	Structural (gray)	SR/MA	na	na	na	trend ↑pituitary in CHR trend ↑pituitary with conv.
Iwashiro et al. 2012 [25]	Structural (gray)	SS	23.6	20	Japan	↓pars triangularis
Ziermans et al. 2012 [51]	Structural (gray)	SS	15.6	43	Netherlands	↓L ant cing, precueus, temp/parietal/occ with conv.
Fusar-Poli et al. 2012 [52]	Structural (gray)	ma	22.5	198	Na AN	↓temporal, limbic prefrontal ↓temporal ant cing, cerebell, insula with conversion
Jung et al. 2012 [53]	Structural (gray)	SS	21.6	16	South Korea	↓Broca’s area
Fusar-Poli et al. 2011 [24]	Structural (gray)	MA	28	896	na	↓R sup temporal, L precuneus, L med frontal, R mid frontal, B parahipp/hipp, ant cingulate ↓R inf frontal, sup. temp in conv.
Mechelli et al. 2011 [54]	Structural (gray)	SS	23.3	182	England Germany Switzerland Australia	↓frontal ↓L parahippocampal in conv.
Smieskova et al. 2010 [55]	Structural (gray)	SR/MA	na	385*	na	↓prefront, cing, insula, cerebel in conv.
Koutsouleris et al. 2009 [27]	Structural (gray)	SS	25.1	45	Germany	↓prefront, orbitofr, limbic, cerebel ↓lat and med temporal in conv.
Sun et al. 2009 [30]	Structural (gray)	SS	19.5	35	Australia	↓R prefrontal in conv.
Takahashi et al. 2009 [56]	Structural (gray)	SS	20.2	97	Australia AN	↓insula with conv.
Takahashi et al. 2009 [31]	Structural (gray)	SS	20.2	35	Australia AN	↓planum polare/temporale in conv.
Ziermans et al. 2009 [35]	Structural (gray)	SS	15.8	54	Netherlands	none
Borgwardt et al. 2007 [20]	Structural (gray)	SS	na	12	Switzerland	↓post. cing, precuneus, paracent, L sup parietal, and ↑L parietal/post temp in conv.
Velakoulis et al. 2006 [34]	Structural (gray)	SS	20.1	135	Australia	none in hippocampus and amygdala
Pantelis et al. 2003 [29]	Structural (gray)	SS	19.3	75	Australia	↓temporal, inf. frontal, cingulate ↓L parahipp, fusiform, orbitofrontal, cerebellar, cingulate with conv.

ACC = anterior cingulate cortex; AN = antipsychotic naïve; BG = basal ganglia; conv = conversion to psychosis; cerebell = cerebellum; cing = cingulate; DLPFC = dorsolateral prefrontal cortex; ENIGMA = Enhancing Neuro Imaging Genetics through Meta-Analysis; hipp = hippocampus; inf = inferior; L = left; lat = lateral; MA = meta-analysis; med = medial; na = not available; mPFC = medial prefrontal cortex; MTL = medial temporal lobe; NAPLS = North American Prodrome Longitudinal Study; n.s. = not significant; parahipp = parahippocampus; orbitofr = orbitofrontal; prefront = prefrontal; R = right; SFG = superior frontal gyrus; SR = systematic review; SS = single study; sup = superior; temp = temporal; triang = triangularis; vol = volume; w = with.

* across multiple modalities

a includes high-risk groups other than CHR; When CHR converters and non-converters are reported separately, the displayed mean age is from non-converters.

**Table 2.** White matter neuroimaging studies in CHR and psychosis conversion.

Author/year	Modality	Study Type	Age range (mean)	Case *n*	Country, Other	Abnormalities
Waszczuk et al., 2022 [64]	Diffusion	SS	25.1	12	Poland	no group difference in CHR
Smigielski et al., 2022 [65]	Diffusion	SS	20.9	37	Switzerland	↓FA in splenium of CC ↓FA in CC, corona radiata, motor/sensory tracts with conv.
Waszczuk et al., 2021 [66]	Diffusion	SR	na	881	na	subtle changes usually esp. in SLF, ILF, IFOF
Nagele et al., 2021 [67]	Diffusion	SS	21.3	30	Germany	no CHR group difference Widespread ↓FA prior to conv
Merritt et al., 2021 [42]	Diffusion	SR	na	2473^a^	na	No normal longitudinal volume and FA increase Decreasing volume and FA w conv.
Kristensen et al., 2021 [68]	Diffusion	SS	24	110	Denmark	Global FA predicted conversion
Fitzsimmons et al. 2020 [61]	Diffusion	SS	21.1	20	US	Cingulum ↓FA↑RD↑trace in CHR
Tomyshev et al. 2019 [33]	Diffusion	SS	20.4	30	Russia males only	↑RD in L ATR
Krakauer et al. 2018 [69]	Diffusion	SS	24.1	30	Denmark	↑FA in L SLF after 12 months
Krakauer et al. 2017 [60]	Diffusion	SS	23.7	45	Denmark	Widespread ↓FA↑RD↓AD in CHR
Rigucci et al., 2016 [70]	Diffusion	SS	21.3	27	Italy	↓FA in CC, L SLF, L ILF, forceps ↑RD in CC, ATR, cingulum
Bakker et al., 2016 [71]	Diffusion	SS	24.3	23	Netherlands	no FA group difference ↑MD, RD in CC, ATR, cortical fasciculi in CHR
Vijayakumar et al., 2016 [72]	Diffusion	SR	na	na	na	↓fronto-temporal and fronto-limbic connections, including SLF, uncinate fasc, cingulum and CC
Katagiri et al. 2015 [73]	Diffusion	SS	na	41	Japan	↓FA in region of CC in CHR
Klauser et al. 2015 [26]	Structural (white)	SS	21.5	69	Singapore	none
Schmidt et al., 2015 [74]	Diffusion	SS	25.4	28	Switzerland	↑FA in SLF, uncinate, R ATR
Von Hohenberg et al. 2014 [63]	Diffusion	SS	20.6	28	US	↑MD in regions of R SLF, corona radiata, CC in CHR
Ziermans et al. 2012 [51]	Structural (white)	SS	15.6	43	Netherlands	↓cerebal wm increase ↓cerebal wm increase with conv.
Carletti et al. 2012 [75]	Diffusion	SS	23.4	32	England	Widespread ↓FA in CHR Widespread ↓FA with conv
Bloemen et al. 2010 [76]	Diffusion	SS	18.9	37	Netherlands	↓FA in R putamen and L SLF with conv. ↑FA in L MTL with conv.
Peters et al. 2010 [77]	Diffusion	SS	21.2	17	Netherelands male only	no baseline group differences
Karlsgodt et al. 2009 [62]	Diffusion	SS	17	36	US	SLF ↓FA in CHR ↓FA in MTL and ILF predicted social dysfunction
Witthaus et al. 2008 [59]	Structural (white)	SS	25.1	30	Germany	↓L sup temp lobe
Walterfang et al. 2008 [78]	Structural (white)		20.2	100	Australia AN	no CC abnormality in CHR ↓ant genu of CC with conv.

AN = antipsychotic naïve; ATR = anterior thalamic radiation; BG = basal ganglia; CC = corpus callosum; CHR = clinical high risk (for psychosis); conv = conversion to psychosis; DLPFC = dorsolateral prefrontal cortex; IFOF = inferior front-occipital fasciculus; IPL = inferior parietal lobule; ILF = inferior longitudinal fasciculus; FA = fractional anisotropy; fasc = fasiculus/fasciculi; FC = functional connectivity; M = mean; MA = meta-analysis; MD = mean diffusivity; na = not available; MFG = medial frontal gyrus; mPFC = medial prefrontal cortex; MRS = magnetic resonance spectroscopy; MTL = medial temporal lobe; NAPLS = North American Prodrome Longitudinal Study; n.s. = not significant; PET = positron emission tomography; QR = quantitative review; RD = radial diffusivity; SFG = superior frontal gyrus; SLF = superior longitudinal fasciculus; SR = systematic review; SS = single study; STG = superior temporal gyrus; STS = superior temporal sulcus; sup = superior; temp = temporal; w = with; wm = white matter.

a includes high-risk groups other than CHR.

**Table 3.** Functional connectivity studies in CHR and psychosis conversion.

Author/year	Modality	Study Type	Age Rang (mean)	Case *n*	Country, other	Abnormalities
Sasabayashi et al., 2023 [85]	FC	SS	17.8	31	Japan	↑connectiv bw DMN and occipital
Fryer et al., 2022 [86]	FC	SS	20.3	45	US	↑connectiv w middle temporal ↓connectiv w cerebell and thalamus
Nogovitsyn et al., 2022 [87]	FC	SS	16.8	51	Canada	↑connectivity between cerebellum, somatomotor network and middle temporal
Bulbul et al., 2022 [88]	FC	SS	20.2	20	Turkey	↑connectiv bw DMN and occipital ↓connectiv bw DMN and DAN
Osborne et al., 2021 [89]	FC	SS	18.9	56	US	auditory temporal accuracy deficits associated with abnormal connectiv bw ant cerebellum and striatum
Del Fabro et al., 2021 [90]	FC	MA	na	810	na	↓connectivity in salience network in CHR
Cao et al. 2020 [91]	FC	SS	12–35	72	US/Canada NAPLS	efficiency in DMN and ↑node diversity across all networks with conv.
Collin et al. 2020 [92]	FC	SS	19.2	137	China	Outcomes predicted by DMN and FPN within network connectivity, and multiple between network connectivities
Collin et al. 2020 [93]	FC	SS	18.7	158	China	abnormal modular connectome organization (STG, ACC) with conv.
Li et al., 2019 [94]	FC	SS	24.6	24	China	↓connectivity bw post insula/somatosensory and bw ant insula/putamen
Zhu et al., 2019 [95]	FC	SS	22	74	China	↓functional asymmetry in L thalam
Du et al., 2018 [96]	FC	SS	20.4	53	US	Dynamic FC impairments in cerebellum, frontal, thalamus, temporal, and between superior frontal and calcarine cortex in dominant state
Bang et al., 2018 [97]	FC	SS	20.4	23	South Korea	↓connectiv bw cerebellum with L preSMA and R anterior PFC
Mennigen et al., 2018 [98]	FC	SS	20.4	53	US	↓neural dynamism in all domains
Cao et al. 2018 [82]	FC	SS	12–35	182	US/Canadas NAPLS	↑connectiv in cerebello-thalamo-cortical circuitry in CHR and with conv.
Pelletier-Baldelli et al., 2018 [99]	FC	SS	19.1	31	US	↓dynamic FC conn involving salience net/DMN w sensory/motor/cognitive regions
Colibazzi et al. 2017 [100]	FC	SS	21	51	US	Abnormal patterns of temporal to thalamus connectivity ↑ACC and frontal conn with symptom severity
Bernard et al., 2017 [101]	FC	SS	18.7	26	US	Abnormal cerebello-thalamo-cortical network connectiv with conv.
Wang et al. 2016 [81]	FC	SS	21.5	34	China AN	↑conn R cerebell and post cing ↑conn cerebell and L sm prefront
Anticevic et al. 2015 [80]	FC	SS	12–35	243	US/Canada NAPLS	↓conn in thalamo-cortico-cerebell circuitry in CHR and with conv. ↑conn bw thalamus and sensorimotor areas with conv.
Pelletier-Baldelli et al., 2015 [102]	FC	SS	18.9	36	US	↓conn bw salience network and medial PFC (of DMN)
Yoon et al., 2015 [103]	FC	SS	20.8	41	South Korea	↑conn bw planum temp to DLPFC ↓conn bw R Heschl’s and ACC
Fryer et al. 2013 [83]	FC	SS	17	32	US	↓suppression of DMN with cognitive load
Gee et al., 2012 [84]	FC	SS	18.8	20	US/Canada NAPLS	↓amygdala-prefrontal conn
Allen et al. 2012 [104]	FC	SS	24.2	41	England	↑midbrain-PFC connectivity w conv.
Jung et al., 2012 [53]	FC	SS	21.6	16	South Korea	↓connectivity bw Broca and frontal
Lord et al. 2012 [105]	FC	SS	24.5	37	England	no group diff in global network org ↓topographical centrality of ACC with conv.
Shim et al., 2010 [106]	FC	SS	20.8	19	South Korea	↑connectivity in DMN ↓anti-correlations bw post cingulate and task areas

ACC = anterior cingulate cortex; AN = antipsychotic naïve; ant = anterior; ATR = anterior thalamic radiation; BG = basal ganglia; bw = between; CC = cerebell = cerebellum; corpus callosum; cing = cingulate; conn/connective = connectivity; conv = conversion to psychosis; DAN = dorsal attention network; diff = differenece; DLPFC = dorsolateral prefrontal cortex; DMN = default mode network; FPN = frontoparietal network; MA = meta-analysis; na = not available; MFG = medial frontal gyrus; mPFC = medial prefrontal cortex; MTL = medial temporal lobe; NAPLS=North American Prodrome Longitudinal Study; n.s. = not significant; QR = quantitative review; SFG = superior frontal gyrus; SR = systematic review; SS = single study; SLF = superior longitudinal fasciculus; sm = superior-medial; SMA = supplementary motor area; STG = superior temporal gyrus; STS = superior temporal sulcus; w = with.

**Table 4.** Other functional neuroimaging studies in CHR and psychosis conversion.

Author/year	Modality	Study Type	Age Rang (mean)	Case *n*	Country, other	Abnormalities
Zeng et al., 2023 [112]	Functional	MA	23.3	318	na	↑activation during reward anticipation in med PFC, ACC ↓activation during reward anticipation in putamen, parahipp, cerebell
Luna et al. 2022 [58]	Functional	SR/MA	29.3	1441	na	↓activation in sup frontal, R inf frontal, R precuneus (n.s.)
Lukow et al., 2021 [113]	Functional	SR/MA	na	na	na	normal emotion activation in CHR
Dutt et al. 2015 [107]	Functional	QR	na	na (22 studies)	na	Dysfunctional R IPL, L MFG, L STG and R SFG in CHR
Karlsgodt et al. 2014 [114]	Functional	SS	16.9	20	US	age-associated frontal activation with conv.
Fusar-Poli, 2012 [52]	Functional	MA	na	na	na	↓activation in L inf frontal and cluster in med frontal, sup frontal and L ACC
Allen et al. 2012 [104]	Functional	SS	24.2	41	England	↑activation in PFC, brainstem, and L hippocampus with conv.
Choi et al. 2012 [109]	Functional	SS	21.6	21	South Korea	↓activation of frontoparietal with task encoding
Sabb et al. 2010 [115]	Functional	SS	16.8	40	US	↑activity in language-associated regions in CHR ↑activity in STG, L IFG and caudate with conv.
Smieskova et al. 2010 [55]	Functional	SR/MA	na	385*	na	↓activation of prefrontal
Crossley et al. 2009 [108]	Functional	SS	na	16	Australia AN	↑activation of STG w *n*-back task
Modinos et al. 2018 [116]	ASL	SS	21.8	36	England	↓correlation between L hipp rCBF and mPFC GABA with conv.
Allen et al. 2018 [117]	ASL	SS	22.6	77	England	↑rCBF of R hippo and BG in CHR
Kindler et al. 2018 [118]	ASL	SS	19.3	29	Switzerland	↓ PFC rCBF and ↑striatal rCBF
Hubl et al., 2018 [119]	ASL	SS	19.3	29	Switzerland	↑striatal CBF w exec functioning deficits
Allen et al. 2016 [111]	ASL	SS	22.4	52	England	↑rCBF in hippoc, BG and midbrain in CHR ↓ rCBF in hippoc, ventral striatum with improvement

AN = antipsychotic naïve; ASL = asrterial spin labeling; BG = basal ganglia; CC = corpus callosum; cerebell = cerebellum; CHR = clinical high risk (for psychosis); conv = conversion to psychosis; DLPFC = dorsolateral prefrontal cortex; GABA = gamma aminobutyric acid; inf = inferior; MA = meta-analysis; MD = mean diffusivity; na = not available; med = medial; MFG = medial frontal gyrus; mPFC = medial prefrontal cortex; MTL = medial temporal lobe; NAPLS = North American Prodrome Longitudinal Study; n.s. = not significant; parahipp = parahippocampus; PFC = prefrontal cortex; rCBF = regional cerebral blood flow; SFG = superior frontal gyrus; SR = systematic review; SS = single study; SLF = superior longitudinal fasciculus; STG = superior temporal gyrus; STS=superior temporal sulcus.

**Table 5.** Positron emission tomography (PET) studies in CHR and psychosis conversion.

Author/year	Modality	Study Type	Age Rang (Mean)	Case *n*	Country, other	Abnormalities
Di Biase et al. 2017 [121]	PET	SS	20.7	10	Australia	No ^11^C-(*R*)-PK11195-binding (microglial activity) abnormality
Hafizi et al. 2017 [122]	PET	SS	21.2	22	Canada	No abnormality in microglial activation using the TSPO radioligand, [^18^F]FEPPA
Bloomfield et al. 2016 [123]	PET	SS	24.3	14	US	↑[(11)C]PBR28 binding ratio in gray matter in CHR
Kang et al., 2014 [124]	PET	SS	19	11	South Korea	↓binding of GABA-A/benzodiazepine receptors in R caudate
Egerton et al. 2013 [125]	PET	SS	22.7	26	England	↑dopamine synthesis capacity in striatum
Allen et al. 2012 [104]	PET	SS	24.2	21	England	↓dopaminergic function in brainstem ([^18^F]-DOPA) with conv.
Howes et al. 2011a [126]	PET	SS	25	20	England	Increasing ([^18^F]-DOPA) uptake in sensorimotor striatum w conv.
Howes et al. 2011b [127]	PET	SS	23.8	30	England	↑dopamine synthesis capacity ([^18^F]-DOPA) in the striatum with conv.
Fusar-Poli et al. 2010 [128]	PET	SS	26.6	20	England	↑Ki for [(19)F]fluordopa in associative striatum in CHR ↓ task activation in R mid frontal, L superior parietal
Howes et al. 2009 [129]	PET	SS	25.6	24	England	↑striatal (18)F-dopa uptake
van Hooijdonk et al., 2022 [130]	PET/SPECT/NM-MRI	SR	na	na	na	striatal D_2/3_ receptor availability normal, ↑striatal dopamine synthesis capacity in some CHR
Howes et al., 2020 [131]	PET/MRS	SS	23.0	51	England	Dopamine synthesis capacity did not predict conv., only symptoms
Fusar-Poli et al., 2007 [132]	PET/SPECT/MRS	SR/MA	na	na	na	abnormalities in prefrontal, ACC, BG, hippocampus and cerebellum

ACC = anterior cingulate cortex; AN = antipsychotic naïve; ATR = anterior thalamic radiation; BG = basal ganglia; CC = corpus callosum; conv = conversion to psychosis; DLPFC = dorsolateral prefrontal cortex; DOPA = dihidryoxyphenylalanine; GABA = gamma aminobutyric acid; MA = meta-analysis; na = not available; MFG = medial frontal gyrus; mPFC = medial prefrontal cortex; MRS = magnetic resonance spectroscopy; MTL = medial temporal lobe; NAA = *N*-acetyl-aspartate; NAPLS = North American Prodrome Longitudinal Study; n.s. = not significant; NM-MRI = neuromelanin-sensitive magnetic resonance imaging; PET = positron emission tomography; SFG = superior frontal gyrus; SPECT = single photon emission computed tomography; SR = systematic review; SS = single study; SLF = superior longitudinal fasciculus; STG = superior temporal gyrus; STS = superior temporal sulcus; sup = superior; TSPO = translocator protein; w = with.

**Table 6.** Magnetic resonance spectroscopy (MRS) studies in CHR and psychosis conversion.

Author/year	Modality	Study Type	Age Rang (Mean)	Case *n*	Country, other	Abnormalities
Fusar-Poli et al., 2007 [132]	PET/SPECT/MRS	SR/MA	na	na	na	abnormalities in prefrontal, ACC, BG, hippocampus and cerebellum
Romeo et al., 2020 [139]	MRS	MA	na	na	na	↑Glx in mPFC, BG. ↑myo-inositol in DLPFC
Whitehurst et al., 2020 [140]	MRS	SR/MA	na	na	na	↓NAA in hippocampus in CHR
Wenneberg et al., 2020 [141]	MRS	MA	na	243	na	↓thalamic glutamate in CHR
Wang et al., 2020 [142]	MRS	MA	na	na	na	↑Cr in mPFC ↓NAA, Cr and Glx in thalamus ↑ml in DLPFC
Bossong et al. 2019 [143]	MRS	SS	22.4	86	England	↑hippocampal glutamate, myo-inositol, and Cr in conv.
Shakory et al., 2018 [144]	MRS	SS	21	25	Canada	↓hippocampal Glx
de la Fuente-Sandoval et al. 2013 [145]	MRS	SS	20.3	19	Mexico	↑striatal glutamate in conv.
Egerton et al. 2014 [146]	MRS	SS	23.3	75	England	↓thalamic glutamate in non-remission
Jessen et al. 2006 [137]	MRS	SS	27	19	Germany	↓NAA/Cr and NAA/Cho in L frontal and NAA/Cr in ACC ↑Cho/Cr and ↓NAA/Cho w conv.
Wood et al. 2003 [138]	MRS	SS	19.5	30	Australia	↑Cho/Cr and ↓NAA/Cho in DLPFC in CHR

ACC = anterior cingulate cortex; AN = antipsychotic naïve; ATR = anterior thalamic radiation; BG = basal ganglia; CC = corpus callosum; Cho = choline; CHR = clinical high risk (for psychosis); conv = conversion to psychosis; Cr = creatinine; DLPFC = dorsolateral prefrontal cortex; Glx = combined glutamine + glutamate; MA = meta-analysis; na = not available; mI = myo-inositol; MFG = medial frontal gyrus; mPFC = medial prefrontal cortex; MRS = magnetic resonance spectroscopy; MTL=medial temporal lobe; NAA = *N*-acetyl-aspartate; n.s. = not significant; PET = positron emission tomography; SFG = superior frontal gyrus; SPECT = single photon emission computed tomography; SR = systematic review; SS = single study; SLF = superior longitudinal fasciculus; STG = superior temporal gyrus; STS = superior temporal sulcus.; w = with.

## Data Availability

The dataset of the study is available from the authors upon reasonable request.

## ABBREVIATIONS

ASL: arterial spin labeling
CHR: clinical high-risk (for psychosis)
DLPFC: dorsolateral prefrontal cortex
DMN: default mode network
FA: fractional anisotropy
ICV: intracranial volume
IFG: inferior frontal gyrus
IPL: inferior parietal lobule
MD: mean diffusivity
MFG: middle frontal gyrus
medFG: medial frontal gyrus
MRS: magnetic resonance spectroscopy
MTG: middle temporal gyrus
PFC: prefrontal cortex
rCBF: regional cerebral blood flow
SFG: superior frontal gyrus
SLF: superior longitudinal fasciculus
STG: superior temporal gyrus
SVM: support vector machine
vlPFC: ventrolateral prefrontal cortex
